# Clinical and microbiological characterization of sepsis and evaluation of sepsis scores

**DOI:** 10.1371/journal.pone.0247646

**Published:** 2021-03-04

**Authors:** Andre Fuchs, Tafese Beyene Tufa, Johannes Hörner, Zewdu Hurissa, Tamara Nordmann, Matthias Bosselmann, Sileshi Abdissa, Abebe Sorsa, Hans Martin Orth, Björn-Erik Ole Jensen, Colin MacKenzie, Klaus Pfeffer, Achim J. Kaasch, Johannes G. Bode, Dieter Häussinger, Torsten Feldt

**Affiliations:** 1 Department of Gastroenterology, Hepatology and Infectious Diseases, University Hospital Düsseldorf, Heinrich-Heine-University, Düsseldorf, Germany; 2 Hirsch Institute of Tropical Medicine, Asella, Ethiopia; 3 College of Health Sciences, Arsi University, Asella, Ethiopia; 4 Institute of Medical Microbiology and Hospital Hygiene, University Hospital Düsseldorf, Heinrich-Heine-University, Düsseldorf, Germany; 5 Institute of Medical Microbiology and Hospital Hygiene, University Hospital Magdeburg, Otto-von-Guericke-University, Magdeburg, Germany; Hospital Universitari Bellvitge, SPAIN

## Abstract

**Background:**

Despite the necessity of early recognition for an optimal outcome, sepsis often remains unrecognized. Available tools for early recognition are rarely evaluated in low- and middle-income countries. In this study, we analyzed the spectrum, treatment and outcome of sepsis at an Ethiopian tertiary hospital and evaluated recommended sepsis scores.

**Methods:**

Patients with an infection and ≥2 SIRS criteria were screened for sepsis by SOFA scoring. From septic patients, socioeconomic and clinical data as well as blood cultures were collected and they were followed until discharge or death; 28-day mortality was determined.

**Results:**

In 170 patients with sepsis, the overall mortality rate was 29.4%. The recognition rate by treating physicians after initial clinical assessment was low (12.4%). Increased risk of mortality was significantly associated with level of SOFA and qSOFA score, Gram-negative bacteremia (in comparison to Gram-positive bacteremia; 42.9 versus 16.7%), and antimicrobial regimen including ceftriaxone (35.7% versus 19.2%) or metronidazole (43.8% versus 25.0%), but not with an increased respiratory rate (≥22/min) or decreased systolic blood pressure (≤100mmHg). In Gram-negative isolates, extended antimicrobial resistance with expression of extended-spectrum beta-lactamase and carbapenemase genes was common. Among adult patients, sensitivity and specificity of qSOFA score for detection of sepsis were 54.3% and 66.7%, respectively.

**Conclusion:**

Sepsis is commonly unrecognized and associated with high mortality, showing the need for reliable and easy-applicable tools to support early recognition. The established sepsis scores were either of limited applicability (SOFA) or, as in the case of qSOFA, were significantly impaired in their sensitivity and specificity, demonstrating the need for further evaluation and adaptation to local settings. Regional factors like malaria endemicity and HIV prevalence might influence the performance of different scores. Ineffective empirical treatment due to antimicrobial resistance is common and associated with mortality. Local antimicrobial resistance statistics are needed for guidance of calculated antimicrobial therapy to support reduction of sepsis mortality.

## 1. Introduction

### 1.1. Introduction

Infectious diseases remain a leading cause for morbidity and mortality in low and middle income countries, in particular in sub-Saharan Africa (SSA) [1]. Here, in contrast to the declining mortality due to Human Immunodeficiency Virus (HIV) infection, malaria and tuberculosis in the past decade, the mortality attributed to severe bacterial infections plays an increasing role. The intensive and longstanding programs to curb the mortality of the above-mentioned diseases were finally successful, whereas bacterial diseases and sepsis have not been in the focus of attention and funding until recently. Furthermore, antimicrobial resistance is increasing and contributing to the rise in mortality in the light of limited treatment options. Regarding sepsis in SSA, awareness is limited [2] and the WHO Global Burden of Diseases Reports do not list sepsis as specific cause of death. Due to various reasons, diagnostic criteria for recognition and prognostic evaluation of sepsis are often not systematically applied in the clinical routine and it has therefore to be assumed that a high number of cases remain unrecognized and unreported. Therefore, interventions and investigations focusing on sepsis treatment do not cover the full extent of the problem and alone may not be sufficient to solve the problem of high sepsis mortality. Tools for early recognition of sepsis as the SIRS score proved ineffective and SOFA and qSOFA scoring have recently been proposed as clinical tools for sepsis recognition [3]. Early recognition of patients at risk is necessary to allocate limited resources and to achieve improved treatment outcomes in resource-limited countries.

In 2017, the international Surviving Sepsis Campaign (iSSC) published revised guidelines for the diagnosis and treatment of sepsis and septic shock after publication of the Sepsis-3 definition in 2016, a renewed definition of sepsis as life-threatening organ dysfunction caused by a dysregulated host response to an infection [3, 4]. With this publication, the SOFA (Sepsis-related Organ Failure Assessment) score and, for use in adult patients only, the qSOFA (quick SOFA) score were introduced for early recognition of organ dysfunction and prognostic assessment of sepsis (see Tables 1 and 2). However, these scores have been extracted from large cohorts of patients, which have mostly been investigated in high income countries without major resource limitations in the health care system and with no relevant causes for sepsis apart from bacterial infections [5, 6]. The diagnostic and prognostic value of these sepsis scores have yet to be evaluated for the setting of SSA, where tropical diseases like malaria substantially contribute to the total disease burden [7].

**Table 1:** SOFA score.

SOFA score	0	1	2	3	4
**Respiration**					
PaO_2_/FIO_2_ (mmHg)	>400	<400	<300	<200	<100
SaO_2_/FIO_2_		221–301	142–220	67–141	<67
**Coagulation**					
Platelets (x10^3^/mm^3^)	>150	<150	<100	<50	<20
**Liver**					
Bilirubin (mg/dl)	<1.2	1.2–1.9	2.0–5.9	6.0–11.9	>12.0
**Cardiovascular**					
Hypotension (MAP in mmHg)	No hypotension	<70			
Dopamine or norepinephrine (μg/kg/min)			Dopamine ≤ 5 or dobuta-mine (any)	Dopamine > 5 or norepi-nephrine ≤ 0.1	Dopamine > 15 or norepi-nephrine > 0.1
**CNS**					
Glasgow Coma Scale	15	13–14	10-Dec	06-Sep	<6
**Renal**					
Creatinine (mg/dl) urine output (ml/d)	<1.2	1.2–1.9	2.0–3.4	3.5–4.9 or <500	>5.0 or <200

A score ≥2 is considered positive, and associated with an increased mortality risk according to [8]

**Table 2:** qSOFA score.

Criteria	Positive Finding
**Respiratory rate**	>22 /min
**Altered cognition (GCS)**	<15
**Systolic blood pressure**	≤100 mmHg

Each criterion is rated with 1 point if within given range, a score ≥2 is considered positive, and associated with an increased mortality risk according to [9]

The availability of systematic data regarding the epidemiology of sepsis, the spectrum of causing organisms and their resistance patterns, as well as the evaluation of management and outcome of patients with sepsis in SSA is limited. The impact of antimicrobial resistance (AMR) and malaria epidemiology, as well as optimal treatment modalities for sepsis on the clinical outcome remain unclear [10]. Available data on resistance patterns of Gram-negative isolates from countries in SSA is alarming. A severe bacterial infection caused by multi-resistant bacteria has a poor prognosis considering the limited choice of antimicrobial treatment regiments [11, 12].

### 1.2. Sepsis in sub-Saharan Africa

Because of the uncertainties described above, established guidelines for treatment of sepsis from developed countries cannot simply be transferred to SSA and treatment of sepsis often remains suboptimal [13, 14]. This problem is intensified by limitations of resources at a local health care level and the lack of specially trained health care workers (HCW), intensive care units, and adequate microbiology laboratories. The sparse existing data indicate that sepsis-associated morbidity and mortality are of great importance in countries across SSA, and even in maximum care facilities in high-income countries, sepsis and septic shock are associated with a high case fatality rate [15]. Therefore, there is an urgent need for improved medical care for patients with sepsis and septic shock especially in SSA [16].

Data from the iSSC suggests that blood culture diagnostics in patients with sepsis is associated with an increased survival rate [17, 18]. In one study performed in Uganda, sepsis was associated with a high mortality rate of >30% and risk factors for mortality were impaired level of consciousness and malnutrition [19]. Another study from Uganda in women with postpartum infections showed extensive AMR in 80% of the isolated Gram-negative bacteria. In the investigated patients, empiric antimicrobial treatment was effective in only 14% of cases [20]. Other investigators also described alarming rates of AMR among bacterial strains isolated from patients with sepsis, and associated risk of failing empiric antimicrobial therapies across different countries in SSA [21–24].

### 1.3. Local situation in Ethiopia

To date, in the Ethiopian setting no specific diagnosis and treatment guidelines for patients with sepsis exist. Scoring systems for early recognition of sepsis patients like the fast and easy applicable qSOFA score [9] are not regularly implemented in standard care. Measured against the international treatment guidelines published by the iSSC, suboptimal patient care with delayed initiation of antimicrobial treatment and insufficient fluid resuscitation is likely. To our knowledge, no systematic study on recognition and treatment of sepsis in adults has been conducted in Ethiopia.

### 1.4. Objectives

This study aims to evaluate the feasibility, reliability and performance of the iSSC-recommended clinical scores SOFA and qSOFA, to investigate recognition and management of sepsis, as well as treatment outcome and risk factors for unfavorable outcome in the setting of a teaching and referral hospital in Central Ethiopia.

## 2. Materials and methods

### 2.1. General information

This cross-sectional prospective observational study was performed for the duration of 10 months (March 2017 to April 2018) at the Asella Teaching and Referral Hospital (ATRH). The ATRH is situated at around 2,400 m above sea level and serves as referral hospital for a population of approximately 4 million people of the Arsi and Bale zones in the eastern Ethiopian highlands.

### 2.2. Ethical considerations

Ethical approval has been obtained by the institutional ethical review board of Arsi University in Asella, Ethiopia, the National Ethical Review Board of the Ethiopian Ministry of Science and Technology in Addis Ababa, Ethiopia, and the Ethics Committee of the Faculty of Medicine, Heinrich-Heine-University, Düsseldorf, Germany. Written informed consent was obtained from all participating patients or legal guardians (if appropriate) before inclusion. The study was conducted according to the principles of the Declaration of Helsinki.

### 2.3. Inclusion

During the study period, patients ≥1 year of age admitted to the ATRH during usual business hours (Monday to Friday) with signs of infection according to the appraisal of the treating physician were screened for SIRS (Systemic Inflammatory Response Syndrome) criteria by the study team (see Table 3). The primary diagnosis documented during the admission process was recorded and could also be of non-infectious nature (e. g. decompensated heart failure), if signs of infection were also present at the same time. White blood cell count (WBC), body temperature, heart rate and respiratory rate were recorded.

**Table 3:** SIRS criteria.

Criteria	Positive finding
**Body temperature**	>38°C or <36°C
**Heart rate**	>90/min
**Respiration rate**	Respiratory rate >20/minute (or PaCO_2_ <32 mmHg)
**White blood cell count**	>12,000/mm^3^ or <4,000/mm^3^ or the presence of >10% immature neutrophils

Each criterion is rated with 1 point if within given range, a score ≥ 2 is considered positive [25].

**Fig 1:**
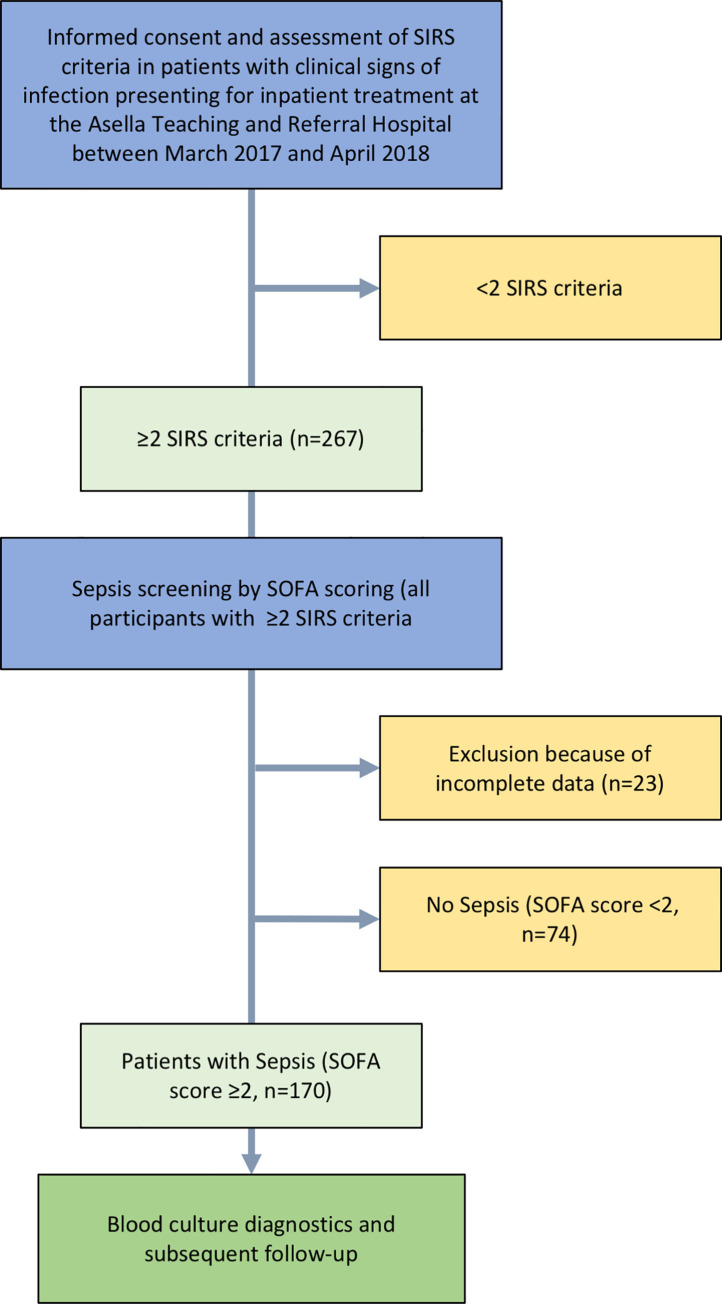
Flow chart of study procedures.

Patients fulfilling ≥2 SIRS criteria in screening investigation by the study team were given the option to take part in the study. Once informed consent was obtained, SOFA score and, in participants ≥18 years, qSOFA score were assessed, peripheral venous blood cultures and a blood sample for blood smear microscopy were drawn. An arterial blood gas analysis was performed to measure lactate levels and PaO_2_ for SOFA score, using the i-STAT®1 analyzer with i-STAT® CG4+ cartridges (Abbott Laboratories, Chicago, USA). Admission diagnoses, vital signs and antimicrobial treatment were recorded. Socioeconomic data and medical history were documented upon admission using a standardized questionnaire. Patients with clinical evidence of an infection and with ≥2 points in the SOFA score were diagnosed with sepsis according to the sepsis-3 definition by the iSSC [26] (see Fig 1).

### 2.4. Follow-up

In patients diagnosed with sepsis according to SOFA score, clinical data and treatment details (e.g. antimicrobial therapy) were recorded daily until discharge, death or referral. After 48 (±24) hours, the SOFA score, SIRS criteria and, in patients ≥18 years, the qSOFA score were reassessed. Information on 28-day mortality was collected from the patient’s file or by phone interview, if patients were already discharged.

### 2.5. Microbiological procedures

One set of commercially available blood culture bottles (aerobe and anaerobe bottles, BacT/ALERT®, bioMerieux, Marcy-l’Étoile, France) was used for blood culturing. Due to limited resources and supplies, no additional blood culture bottles were collected. Regular quality controls for sensitivity were performed by incubation of standard laboratory strains of different bacteria.

All inoculated blood cultures were investigated by Gram staining and light microscopy 24 and 120 hours after inoculation and sub-cultured on Blood, MacConkey and Chocolate Agar in 5% CO_2_ enriched atmosphere if Gram staining showed bacterial growth. Subsequent species identification and antimicrobial sensitivity testing (AST) with standardized antibiotic disks on Mueller Hinton Agar were performed. AST results were interpreted following Clinical & Laboratory Standards Institute (CLSI) guidelines. If Gram staining and subcultures 5 days after inoculation of the blood culture failed to show bacterial growth, the cultures were considered negative.

For quality control, sample colonies of all isolated bacterial strains were exported to the Institute of Medical Microbiology and Hospital Hygiene of the Düsseldorf University Hospital in Düsseldorf, Germany. Species identification was reassessed by using matrix-assisted laser desorption/ionization time-of-flight mass spectrometry (MALDI-ToF MS) and AST were repeated by standardized laboratory procedures. Interpretation of AST results followed EUCAST (European Committee on Antimicrobial Susceptibility Testing) guidelines.

Identification of bacterial resistance genes was performed by quality-controlled molecular biological analysis. After DNA extraction, bacterial strains with suspected production of extended-spectrum beta-lactamases (ESBL) or carbapenemases were investigated by PCR, following the protocols described by Strauß et al. for identification of the beta-lactamase *bla*_CTX-M_, *bla*_SHV_ and *bla*_TEM_ genes and an in-house PCR protocol established for the detection of carbapenemases (*bla*_VIM,_ *bla*_NDM,_ *bla*_KPC,_ *bla*_IMP,_ *bla*_GES,_ *bla*_OXA_) as described by Wendel et al. [27, 28].

In addition, blood smear light microscopy with a 100x oil immersion objective after Giemsa staining was performed from blood samples drawn at study inclusion to investigate for infections caused by hemoparasites.

### 2.6. Data handling and statistical analysis

Statistical analysis of data was performed using IBM SPSS Statistics for Windows, Version 25.0 (IBM Corp., Armonk, NY, USA). Frequency and percent were used to describe qualitative variables; for quantitative variables, mean and standard deviation were used if data were normally distributed. Median and interquartile range (IQR) were used, if data were non-normally distributed, as assessed by Shapiro Wilk test. Pearson’s chi-squared test and Fisher’s exact test were used for the analysis of differences between groups. P-values <0.05 were considered statistically significant.

## 3. Results

### 3.1. Inclusion and general information

**Fig 2:**
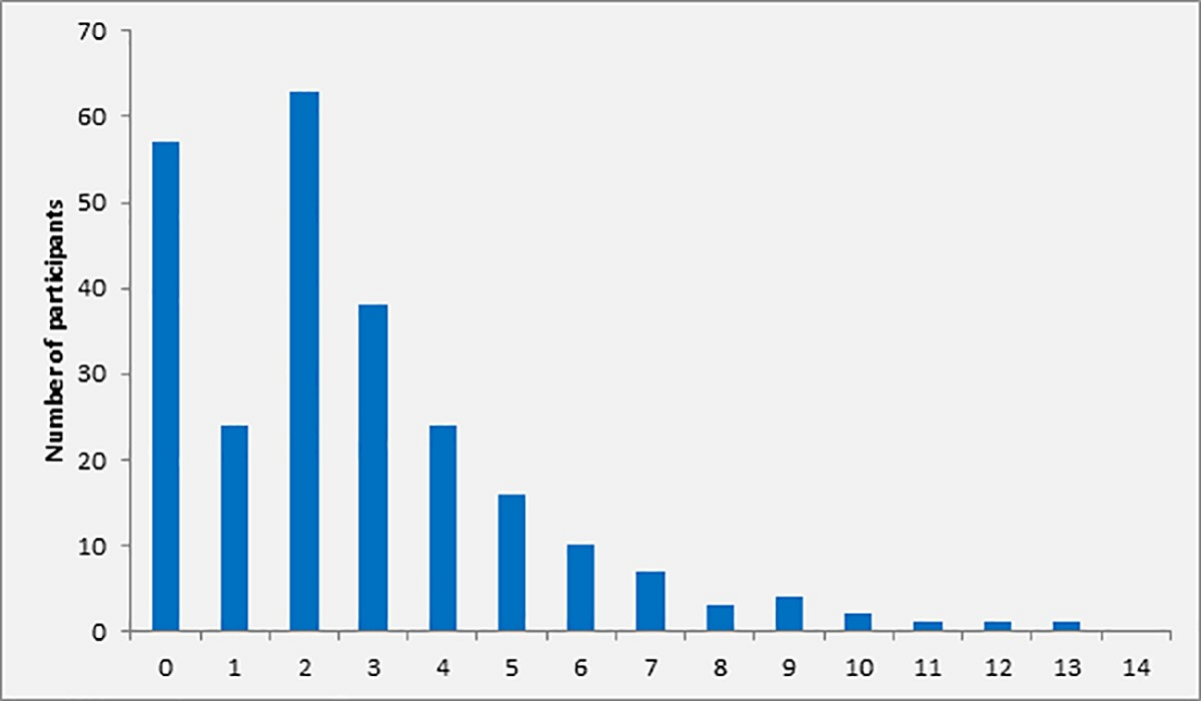
Distribution of SOFA score at screening among participants with ≥2 SIRS criteria (n = 170).

A total number of 267 patients with clinical signs of infection and ≥2 positive SIRS criteria were screened for possible sepsis by SOFA scoring. Twenty-three patients were excluded from further analysis because of incomplete data. In 170 (69.7%, 30 minors [<18 years] and 140 adults [≥18 years]) of the remaining 244 patients eligible for analysis, the SOFA score was ≥2, indicating sepsis. In particular, the SOFA score was <2, 2, 3, 4, and ≥5 in 30.3% (n = 74), 25.8% (n = 63), 15.6% (n = 38), 9.8% (n = 24) and 18.4% (n = 45), respectively (Fig 2). Overall, median SOFA score at screening was 2 (IQR 1–4). Follow-up investigations were performed in patients with sepsis only.

Within the group of patients with sepsis (n = 170), the median age was 30 years (range 1–90 years, IQR 18–46), 48.8% were female and 23.5% were classified as underweight by the treating physician. In total, 30 study participants were minors (i.e. 1–17 years, median age 14 years [IQR 8–16]), and thus excluded from analyses involving the qSOFA score. The remaining 140 adult patients (median age 35 years [IQR 23–50]) were eligible for further analysis involving the qSOFA score.

**Fig 3:**
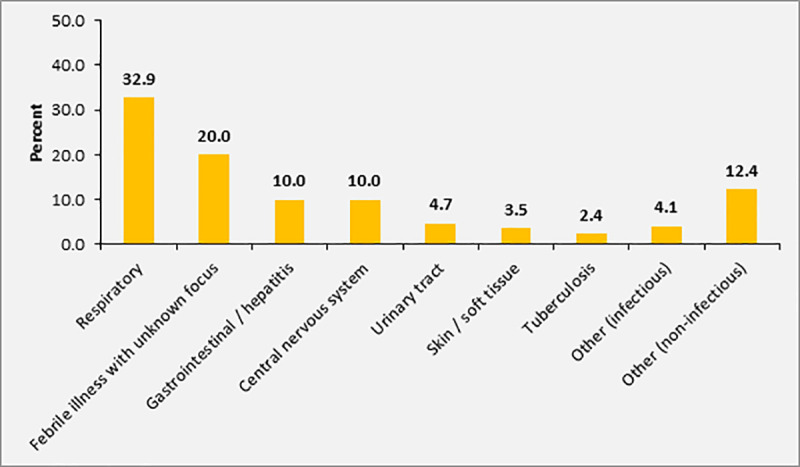
Sources of infection / main diagnosis in patients with SOFA score ≥2 (n = 170).

At least one chronic disease (e.g. diabetes mellitus, hypertension, cardiac insufficiency or HIV infection) was present in 21.8% (n = 37) of participants. In particular, 11.8% (n = 20) were HIV-infected and chronic wounds were present in 15.3% (n = 26) of participants (Table 4). According to initial assessment by the treating physician at admission, 32.9% (n = 56) of the participants with sepsis (SOFA score ≥2) were diagnosed with respiratory infections, 10.0% (n = 17) with central nervous system infections, and 10.0% (n = 17) with gastrointestinal or hepatobiliary infections. In 20.0% (n = 34) of patients, the focus of infection was unclear. The primary diagnosis documented during admission was non-infectious (e. g. cardiovascular complications) in 12.4% of patients (n = 21), who showed concomitant signs of infection according to the appraisal of the treating physician and thus fulfilled the inclusion criteria (Fig 3).

**Table 4:** Demographic and anamnestic characteristics of study population (n = 170).

Variables	% (n)
**Gender**	
Male	51.2 (87)
Female	48.8 (83)
**Age group**	
<18	17.6 (30)
18–65	72.9 (124)
>65	9.4 (16)
**Chronic disease present**	21.8 (37)
**Known HIV infection**	11.8 (20)
**Chronically open wounds**	15.3 (26)
**Nutritional status (approximation)**	
Obese	4.7 (8)
Normal weight	64.1 (109)
Underweight	23.5 (40)
Unknown	7.6 (13)

### 3.2. Outcome of sepsis and risk factors for mortality

**Fig 4:**
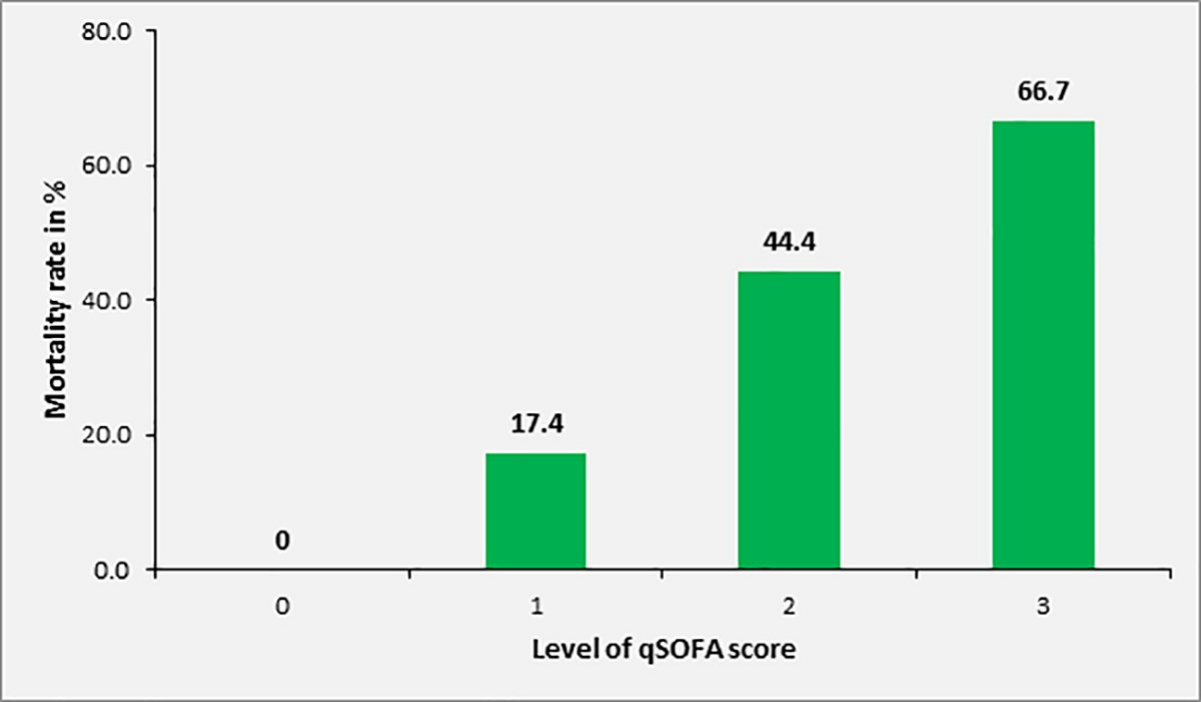
Mortality rate of study participants with sepsis (SOFA ≥2) according to qSOFA score at inclusion (patients ≥18 years, n = 140).

**Fig 5:**
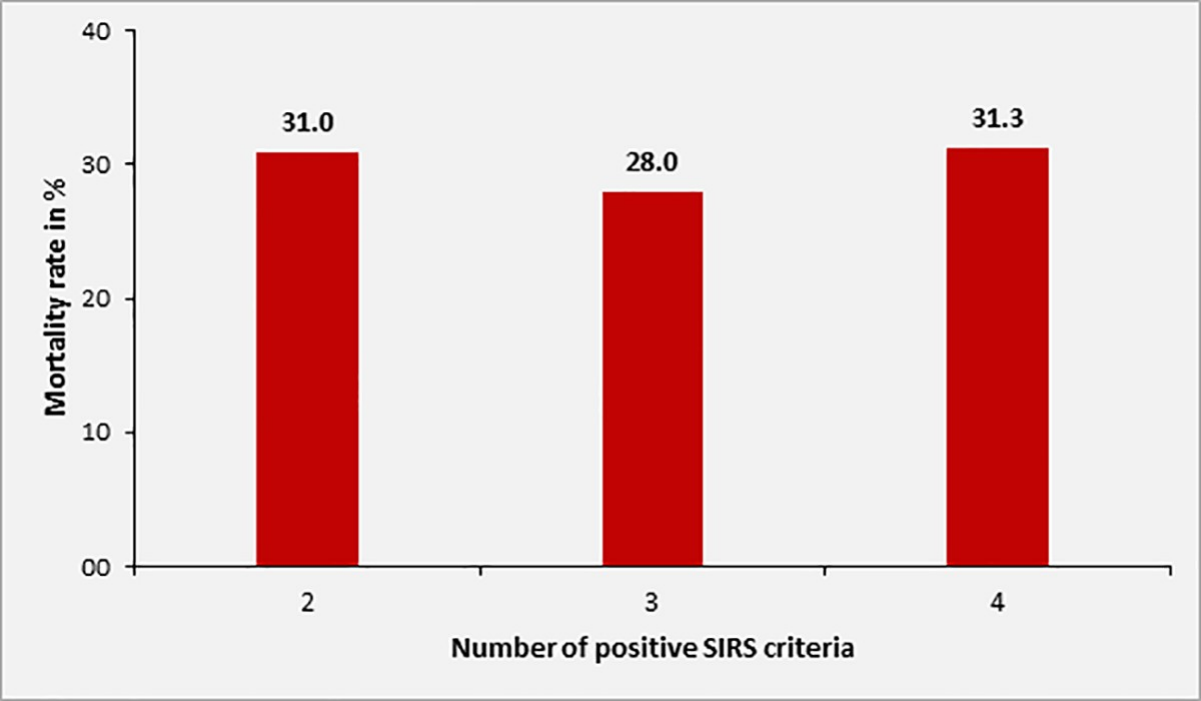
Mortality rate of sepsis patients according to number of positive SIRS criteria at inclusion (n = 170).

Overall, the 28-day mortality of patients with sepsis was 29.4% (n = 40) in 136 patients for whom this information was available. Mortality in patients with SOFA score of 2, 3, 4 and ≥ 5 was 10.4%, 13.8%, 42.1% and 57.5%, respectively. Among adult study participants with sepsis and complete data on 28 day mortality and qSOFA, [n = 110], the 28-day mortality was 0%, 17.4%, 44.4% and 66.7% in patients with qSOFA score of 0, 1, 2 or 3 at inclusion, respectively (Fig 4). Overall, the 28-day mortality in patients with qSOFA ≥2 was 46.7% (28/60). The mortality rate according to SIRS criteria at inclusion was 31.0%, 28.0% and 31.3% in patients with 2, 3 or 4 positive SIRS criteria, respectively (n = 170, Fig 5).

The association between level of SOFA score and increase in mortality rate was statistically significant (p<0.001). Twenty-eight-day mortality was also significantly associated with qSOFA scores ≥2 upon inclusion (p = 0.001), but not with the number of positive SIRS criteria (p = 0.923).

Higher age (>65 years) was associated with increased mortality (60.0% vs. 25.6%, p = 0.022). There was no significant association between 28-day mortality and focus of infection, admission diagnosis, chronic diseases, HIV-status, gender or nutritional status. In addition, there was no significant difference in mortality rate between patients with positive vs. sterile blood cultures upon admission (40.0% vs. 28.1%, p = 0.25).

### 3.3. Follow-up

Data of follow-up assessment of SIRS, SOFA and qSOFA (only participants ≥18 years) 48 hours after admission were available for 53 of 170 participants (31.2%). Of those, 26.4% (14/53) had died before the follow-up investigation. In participants with complete follow-up data, the SOFA-score decreased in 53.8% (n = 21), remained steady in 28.2% (n = 11) and increased in 17.9% (n = 7) of investigated patients. The mortality rate of patients with failure to decrease in level of SOFA score at follow-up was significantly higher compared to patients with decreasing trend (50.0% versus 19.0%, p = 0.041). For SIRS and for qSOFA score (only participants ≥18 years), this association was not statistically significant (Table 5).

**Table 5:** Mortality rate according to the trend of the different scores at follow-up investigation after 48 ± 24 hours.

	Mortality rate (in %)		
Score	Decrease at follow-up	Equal or higher at follow-up	p-value
**SOFA**	19.0	50.0	**0.044**
**qSOFA**	14.3	42.3	0.171
**SIRS**	26.7	37.5	0.367

### 3.4. Recognition of sepsis

According to review of chart documentation, only 12.4% (21/170) of the study participants with sepsis were diagnosed with sepsis or septic shock during the routine clinical assessment by health care workers (HCWs) of the ATRH. In particular, 12.7%, 7.9%, 8.3% and 17.8% of patients with SOFA score of 2, 3, 4 and ≥5, respectively were recognized as patients with sepsis by the hospital’s HCWs. There was no difference in sepsis recognition rate in the subset of adult patients (n = 140) with elevated qSOFA score (≥2) in comparison to those with qSOFA score <2 (14.5% versus 7.8%, p = 0.167). In patients with positive blood cultures, the sepsis recognition rate was 11.1% (2/18) compared with 12.5% (19/152) in patients with negative blood cultures (p = 0.611). There was no difference in mortality rate in patients diagnosed with sepsis compared with those with missed diagnosis of sepsis (28.6 versus 29.5%, p = 0.61).

### 3.5. Respiratory rate and systolic blood pressure, as components of qSOFA score, in adult patients with sepsis

Since respiratory rate and blood pressure are highly age-dependent and considering that the qSOFA score has not been evaluated in pediatric populations, the following analyses were only performed in the subgroup of adult sepsis patients (n = 140). The elevation of the respiratory rate ≥22 /min and the decrease of the systolic blood pressure ≤100 mmHg are decisive components of the qSOFA score, corresponding to one point each. The score is considered positive once two points are reached (see Table 2). At the study site, 2,400 m above sea level, the median respiratory rate was 29.5 /min (IQR 24–36), and 89.3% of participants (n = 125) had a respiratory rate of ≥22 /min. However, there was no significant difference regarding the rate of patients with increased respiratory rate and level of SOFA score (89.7% of patients with SOFA 2–3 and 88.7% of patients with SOFA ≥4; p = 0.528).

The median systolic blood pressure in adult participants was 101.5 mmHg (IQR 90.3–116.0). In 50.0% (n = 70) the systolic blood pressure at inclusion was ≤100 mmHg. There was no significant difference with regard to a reduced systolic blood pressure ≤100 mmHg between patients with SOFA score 2–3 and SOFA score ≥4 (20.5% versus 32.3%; p = 0.83). The mortality rate was not associated with the presence of an elevated respiratory rate (≥22 /min: 31.6%; <22 /min: 41.7%, p = 0.346) or a reduced systolic blood pressure (≤100 mmHg: 29.1%; >100 mmHg: 36.4%, p = 0.271).

### 3.6. Microbiological results

#### 3.6.1. Blood culture isolates

Before blood cultures were drawn, 71.4% (n = 120) of patients had received antimicrobial treatment (see below). The blood cultures yielded positive results in 10.6% (n = 18) and the blood culture positivity rate did not differ between minors and adults (13.3% [4/30] versus 10.0% [14/140], p = 0.395). Coagulase-negative staphylococci (CONS) were isolated in 5.3% (n = 9). Because of a high suspicion for contamination, all blood cultures (n = 9) showing growth of CONS were considered negative. There was no significant difference in blood culture positivity rate in patients with previous administration of antibiotic treatment at the time of sampling compared to patients without antibiotic treatment (9.2% versus 13.7%, p = 0.269).

Gram-positive cocci (GPC) and Gram-negative rods (GNR) were isolated in the same proportion of 44.4% (n = 8) each. In 11.1% of cases (n = 2), *Candida* spp. were isolated. The most common bacterial isolates were *Staphylococcus aureus* (n = 7, 38.9%) and *Escherichia coli* (n = 4, 22.2%). For detailed results of microbiological culturing, see Table 6.

**Table 6:** Isolates from blood cultures at study inclusion.

Isolate	Number	Percentage
**Gram-positive cocci**	8	44.4
*Staphylococcus aureus*	7	38.9
*Enterococcus faecalis*	1	
**Gram-negative rods**	8	44.4
*Escherichia coli*	4	22.2
*Klebsiella pneumoniae*	2	11.1
*Acinetobacter baumannii*	1	
*Pseudomonas aeruginosa*	1	
**Yeasts**	2	11.1
*Candida* spp. (*C*. *albicans* and C. tropicalis)	2	11.1

#### 3.6.2. Blood culture positivity and sepsis scores

There was no significant association between a positive blood culture result and SOFA or qSOFA (only participants ≥18 years) scores, or number of SIRS criteria at inclusion. However, blood cultures from patients with SOFA score ≥5 yielded positive results in 17.8% compared to 8.1% in patients with SOFA score 2–4 (p = 0.201). In our cohort, a SOFA score of ≥5 was significantly associated with Gram-negative bacteremia (11.1% versus 2.4%, p = 0.028), but not with Gram-positive bacteremia or candidemia.

#### 3.6.3. Bacterial isolate and mortality rate

**Fig 6:**
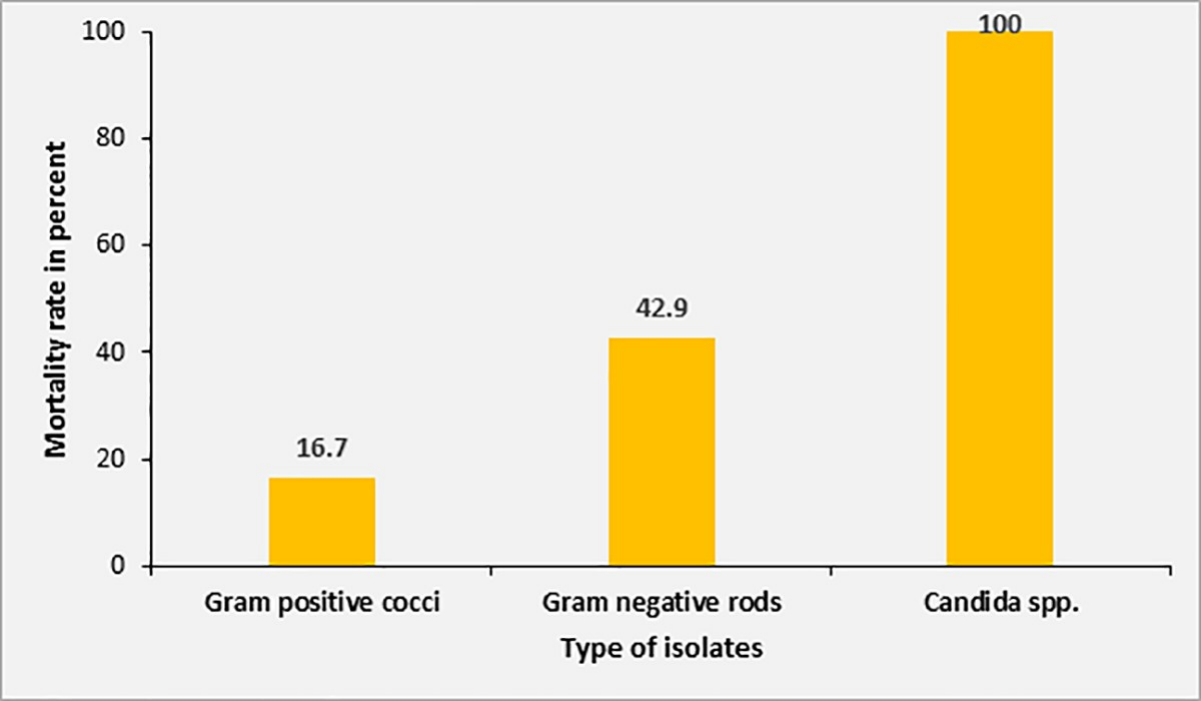
Mortality rate according to different isolates from blood cultures.

Overall, the mortality rate in patients with positive blood culture was 40.0% compared to 28.1% in patients with sterile blood cultures (p = 0.34). The mortality rate was 100% (2/2) in patients with *Candida* sepsis, 42.9% (3/7) in patients with Gram-negative sepsis and 16.7% (1/6) in patients with Gram-positive sepsis (Fig 6). However, none of these differences were significant due to the small numbers in each subgroup.

#### 3.6.4. Blood film investigation

Blood film microscopy from 169 available samples revealed two cases of *Plasmodium vivax* malaria, but no cases of *P*. *falciparum* malaria were detected. Both patients survived until end the end of follow-up.

#### 3.6.5. Antimicrobial resistance

Concerning GPC, 71.4% of the isolated strains of *Staphylococcus aureus* were resistant to co-trimoxazole and 28.6% to clindamycin. None of the isolated *Staphylococcus aureus* strains were *methicillin resistant Staphylococcus aureus* (MRSA). Antimicrobial resistance in the isolated Gram-negative bacterial isolates was much more common. *Enterobacterales* were frequently resistant to aminopenicillins combined with beta-lactamase-inhibitors (83.3%), 3^rd^ generation cephalosporins (66.7%), quinolones (66.7%) and sulfonamides (83.5%). Resistance to aminoglycosides (33.3%) and carbapenems (n = 1) was less frequent but still considerably high. There were only two isolated strains of non-fermenting GNR, of which one displayed a high level of antibiotic resistance due to the production of two different carbapenemases (see below, Table 7). Production of extended-spectrum beta-lactamases was found in 75% (6/8) and carbapenemases (NDM-1 and OXA-51) in 25% (2/8) of the isolated GNR. In only one multi-resistant Gram-negative bacterial strain, none of the resistance genes tested were detected (Table 8). The ESBL-genes *bla*_CTX-M-1_ and *bla*_TEM_ were the most commonly isolated resistance genes.

**Table 7:** Rate of resistance to different antibiotic classes among the bacterial isolates.

	Resistant isolates in % (n)			
Antibiotic class (tested substance)	Gram-positive cocci		Gram-negative rods	
	*S*. *aureus* (n = 7)	*Enterococcus faecalis* (n = 1)	*Enterobacterales* (n = 6)	Non-fermenting Gram-negative rod (n = 2)
**Benzylpenicillin (penicillin G)**	85.7 (6)	1	*nt*	100 (2)
**Isoxazolylpenicillines (oxacillin)**	0 (0)	1	*nt*	100 (2)
**Aminopenicillin (ampicillin or amoxicillin)**	*nt*	1	83.3 (5)	100 (2)
**Aminopenicillin + beta-lactamase-inhibitor (BLI) (clavulanic acid or sulbactam)**	0 (0)	*nt*	83.3 (5)	100 (2)
**Ureidopenicillin (piperacillin)**	*nt*	*nt*	83.3 (5)	100 (2)
**Ureidopenicillin + BLI (piperacillin/tazobactam)**	0 (0)	*nt*	50 (3)	50 (1)
**1^st^ generation cephalosporin (cefazolin or cefalexin)**	0 (0)	*nt*	*nt*	100 (2)
**2^nd^ generation cephalosporin (cefuroxime or cefoxitin)**	0 (0)	*nt*	66.7 (4)	100 (2)
**3^rd^ generation cephalosporins (cefotaxime or ceftazidime)**	0 (0)	*nt*	66.7 (4)	100 (2)
**Carbapenems** **(imipenem or meropenem)**	0 (0)	*nt*	16.7 (1)	50 (1)
**Quinolones (ciprofloxacin or moxifloxacin)**	0 (0)	*nt*	66.7 (4)	100 (2)
**Glycopeptide (vancomycin)**	0 (0)	0 (0)	*nt*	*nt*
**Sulfonamides (trimethoprim-sulfamethoxazole)**	71.4 (5)	100 (1)	83.3 (5)	100 (1)*
**Aminoglycosides (amikacin or gentamicin)**	0 (0)	*nt*	33.3 (2)	50 (1)
**Lincosamides (clindamycin)**	28.6 (2)	*nt*	*nt*	*nt*
**Macrolides (azithromycin or clarithromycin)**	*nt*	*nt*	*nt*	*nt*
**Tetracyclines (tetracycline)**	42.9 (3)	*nt*	*nt*	100 (2)
**Oxazolidinones (Linezolid)**	0 (0)	0 (0)	*nt*	*nt*
**Glycylcyclines (tigecycline)**	0 (0)	0 (0)	0 (0)	50 (1)
**Epoxide antibiotics (fosfomycin)**	0 (0)	*nt*	*nt*	*nt*
**Lipopetide antibiotics (daptomycin)**	0 (0)	*nt*	*nt*	*nt*

Nt, not tested

*, not tested in *Pseudomonas* isolates

**Table 8:** Resistance genes in isolated Gram-negative bacteria.

Bacterial isolate	ESBL			Carbapenemase	
	CTX-M-1	TEM	SHV	NDM-1	OXA-51
*E*. *coli*	**+**	**-**	**-**	**-**	**-**
*E*. *coli*	**+**	**+**	**-**	**-**	**-**
*E*. *coli*	**-**	**+**	**-**	**-**	**-**
*E*. *coli*	**-**	**-**	**-**	**-**	**-**
*K*. *pneumoniae*	**+**	**+**	**+**	**-**	**-**
*K*. *pneumoniae*	**+**	**+**	**+**	**+**	**-**
*Pseudomonas aeruginosa*	**+**	**+**	**-**	**-**	**-**
*Acinetobacter baumannii*	**-**	**-**	**-**	**+**	**+**
**Overall percentage**	**62,5**	**62,5**	**25,0**	**25,0**	**12,5**

ESBL, extended spectrum beta-lactamase

### 3.7. Antimicrobial treatment

**Fig 7:**
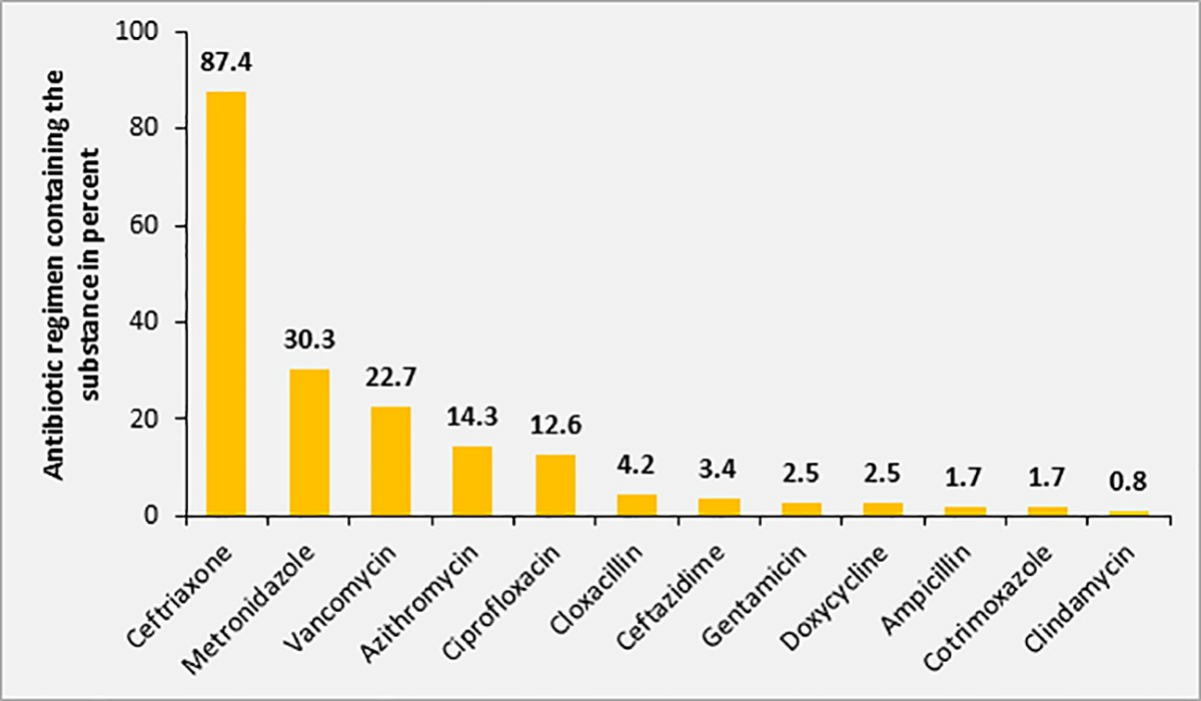
Distribution of antimicrobial substances used for empiric treatment among study participants with sepsis (n = 170).

The majority of patients (70.0%; 119/170) included in this study received antimicrobial treatment at the time of inclusion. All initial antimicrobial treatments were administered empirically. In 74.8% (89/119) of patients receiving antimicrobial treatment, more than one substance was administered. There was no difference in the number of antimicrobial substances used for treatment in patients diagnosed with sepsis by the treating physicians at the ATRH compared to patients without diagnosis of sepsis (≥2 antimicrobial substances, 52.4% versus 52.3%, p = 0.592). In addition, there was no significant difference in the actual SOFA score between patients treated with combined antimicrobial treatment and patients receiving antimicrobial monotherapy (SOFA score ≥4, 46.1% versus 34.6%, p = 0.085). However, the qSOFA score was positive (i.e. ≥2) significantly more often in patients receiving combined antimicrobial therapies compared to patients receiving monotherapy (63.9% versus 44.1%, p = 0.015). Ceftriaxone was the most commonly used substance as part of the antimicrobial treatment regimen (87.4%, n = 104), followed by metronidazole (30.2%, n = 36) plus vancomycin (22.7%, n = 27) (Fig 7).

A significant proportion of patients with sepsis according to SOFA scoring did not receive any initial antibiotic treatment (30.0%, n = 51). Regarding mortality rate, there was no significant difference in study participants with sepsis receiving any antimicrobial treatment compared to those without antibiotic treatment (32.7% [32/98] versus 21.1% [8/38], p = 0.13). Higher mortality rates were significantly associated with an antimicrobial regimen including ceftriaxone (35.7% versus 19.2%, p = 0.03) and metronidazole (43.8% versus 25.0%, p = 0.037), but not with regimen including vancomycin (31.6% versus 29.1%, p = 0.508).

The mortality rate in patients receiving multiple antimicrobials was 34.7% in comparison to 29.6% in patients with single-agent antimicrobial treatment (p = 0.410), although there was no significant difference in the frequency of combined antibiotic treatment regimen regarding the attributed SOFA score (48.8% in patients with SOFA 2–4 versus 62.2% in patients with SOFA score ≥5, p = 0.085).

According to performed AST, at least one of the empirically prescribed antimicrobial substances was tested to be active against the isolated pathogen in only 27.8% (5/18) of cases. Isolated pathogens were susceptible to the administered treatment with ceftriaxone in 44.4% (4/9), to ciprofloxacin in 50% (1/2) and to vancomycin in 33.3% (1/3). Metronidazole was frequently utilized as part of combined treatment regimen; however, no anaerobic bacteria were isolated. All patients, in whom the isolated pathogen was sensitive to at least one of the empirically administered antimicrobials survived. The mortality rate in patients, in whom none of the antibiotics administered was effective against the isolated organism was 55.6% (p = 0.098).

### 3.8. Evaluation of qSOFA as a clinical sepsis score

**Fig 8:**
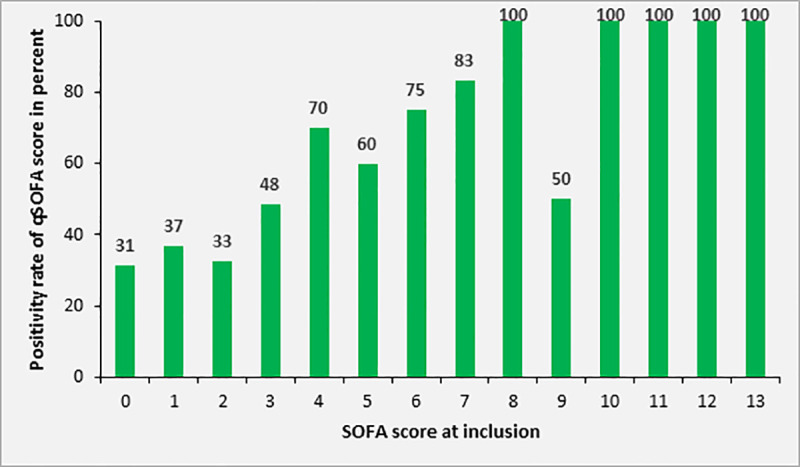
Rate of adult patients with positive qSOFA (≥2) according to SOFA score at study inclusion (n = 140).

At study inclusion, the qSOFA score of adult participants with sepsis (n = 140) was 0, 1, 2 and 3 in 4.3%, 41.4%, 49.3% and 5.0%, respectively. The sensitivity of qSOFA for the recognition of sepsis was 54.3% (76/140) using a SOFA score ≥2 as a gold standard. In patients with a higher SOFA score (i.e. SOFA score ≥4) compared to patients with a SOFA score of 2 or 3, the sensitivity was significantly higher (72.6% versus 39.7%, p<0.001, compare Fig 8).

The specificity of qSOFA for the diagnosis of sepsis was 66.7%; 36 out of 54 adult patients without sepsis had a qSOFA score <2 at study inclusion. The positive and negative predictive value of qSOFA for the diagnosis of sepsis were 80.4% and 36.0%, respectively. The area under the ROC curve was 0.601.

## 4. Discussion

Early recognition of sepsis is essential for optimal treatment and outcome. Therefore, reliable and practical tools are needed in order to achieve this important goal under specific local conditions. The primary objective of this study was to evaluate the feasibility and performance of SOFA and qSOFA scores at the ATRH, a tertiary hospital situated at an altitude of 2,400 m in Central Ethiopia. For preselection of appropriate study participants, SIRS criteria were applied because they are easily and quickly applicable and highly sensitive for recognition of sepsis despite limited specificity [29, 30], followed by SOFA scoring for confirmation of sepsis, as introduced in the sepsis-3 definition by the iSSC [26].

Sepsis was found to be highly prevalent and yet underdiagnosed by the hospital’s medical staff at the study center according to chart documentation, without consideration of the score results. The 28-day mortality rate in our study (29.4%) was considerably higher than mortality rates of 17–18% described from other sepsis cohorts from high income countries [31, 32], but comparable to those described for other low- and middle-income countries [33, 34]. To analyze the quality of sepsis treatment was not part of this investigation. However, restricted diagnostic and therapeutic possibilities, limited capacities of intensive care units and lack of intensive care training are all likely to contribute to the high mortality rate.

As expected, the mortality rate increased significantly with severity of the disease, as indicated by elevation of the SOFA and qSOFA scores, with the latter being calculated in adult participants only. Measurement of the SOFA score, as a gold standard for the diagnosis of sepsis in this study, is complex and requires appropriate laboratory equipment and resources. Although the sensitivity and specificity of the qSOFA score for the diagnosis of sepsis deviated from values published in the literature, it appears a suitable tool to guide the risk-adapted allocation of restricted medical resources, such as intensive care unit capacities or the use of expensive broad-spectrum antimicrobial substances, in resource-limited settings.

The overall recognition rate of sepsis by the treating physicians of the ATRH was strikingly low and sepsis scores are not routinely utilized in the clinical setting. It is noteworthy that there were no significant differences in mortality, rate of positive blood cultures or antibiotic treatment between patients diagnosed with sepsis and patients with a missed sepsis diagnosis. A possible explanation is that these patients may have been recognized as seriously ill by treating physicians without establishing the formal diagnosis of sepsis. However, we are convinced that the correct and standardized detection of sepsis could contribute to an optimized management.

Despite the global emergence of AMR, local AMR statistics and antibiotic susceptibility data for patients in resource-limited settings are largely unavailable in resource-limited settings and thus antimicrobial treatment regimens are mostly prescribed purely empirically. In this study cohort, empirical antimicrobial treatment did not prove beneficial regarding mortality. This observation most likely reflects the extensive AMR, rendering most antimicrobial treatment regimens administered within this cohort ineffective. The overall blood culture positivity rate within the studied population was low. The sensitivity of the blood cultures may be limited, because only one set of blood cultures was investigated, as this is common practice at the study center due to lack of reliable supply and high costs of disposable bottles [35]. There is also widespread reluctance in the local population including HCWs to provide the relatively high blood volume required for optimal sensitivity of blood cultures, due to the common belief that even small amounts of blood loss are associated with a health hazard. Furthermore, a preselection of resistant bacteria might have occurred, since 71.4% of patients had received antibiotics before blood culture sampling.

There was a trend towards a higher mortality rate in patients with bacteremia compared to patients with sterile blood cultures, without reaching statistical significance due to the small case numbers. Among the isolated pathogens, GPC and GNR were isolated in equal proportions, with some cases of candidemia.

Among patients with positive blood cultures, the mortality rate was highest in patients with candidemia and noticeably higher in patients with Gram-negative sepsis compared to patients with Gram-positive sepsis. The probability of receiving effective empirical treatment might play a role for this observation, being higher in Gram-positive pathogens, compared to Gram-negative and fungal pathogens. Due to the high altitude of the study site at 2400 m above sea level, malaria did not play a decisive role as a trigger for sepsis in this cohort in contrast to other sepsis cohorts from SSA. No cases of *P*. *falciparum* malaria were diagnosed and *P*. *vivax* malaria did not lead to mortality.

Among GNR, AMR against multiple antibiotics was common. Regarding GPCs, MRSA was not isolated from any patient. Nevertheless, AMR against sulfonamides and lincosamides, as frequently used antimicrobial substances at the study site, was present in relevant proportions. In GNR, the two ESBL genes *bla*_CTX-M-1_ and *bla*_TEM_ were most frequently detected. Despite the rare application of carbapenems as part of antimicrobial treatment regimens at the ATRH, pathogens carrying carbapenemases (NDM-1 and OXA-51) were isolated in 25% of cases. These findings are consistent with other studies describing the prevalence of ESBL-genes and carbapenemases in Gram-negative isolates from Ethiopia [36, 37].

In our cohort, more than two thirds of the patients had received some kind of empiric antimicrobial treatment at the time of inclusion. Of note, despite the low recognition rate of sepsis and septic shock, almost three quarters of applied regimens comprised at least two substances from different antimicrobial classes. Adult patients with more severe illness according to qSOFA score were more likely to receive a combination antimicrobial therapy. In light of the limited sensitivity of blood cultures and according to susceptibility testing, the empirically administered antimicrobial therapy proved effective only in about one quarter of cases. More than half of the patients (56%) receiving an ineffective antibiotic regimen died, whereas all patients receiving at least one effective substance as part of their treatment survived until end of follow-up. Ceftriaxone was the most commonly used antimicrobial substance but the rate of resistance to ceftriaxone in isolated GNR was high. This finding may explain why the use of ceftriaxone was associated with increased mortality. Likewise, metronidazole treatment was associated with higher mortality and no pathogens susceptible to metronidazole were isolated. However, in the light of the reduced sensitivity of blood cultures, these results must be interpreted with caution.

The specificity of qSOFA score for diagnosing sepsis in the subgroup of adult patients was especially limited in the study population. However, the sensitivity for diagnosis of sepsis or septic shock of 54.3% within our cohort of patients with ≥2 positive SIRS criteria was considerably higher compared to previously published sensitivities ranging between 16.3–32%. On the other hand, the specificity of 66.7% was significantly lower compared to the published range between 96.1–98% [38–40]. In the light of the high proportion of patients with a markedly elevated respiratory rate, the high altitude of the study center with correspondingly lower partial pressure of oxygen might potentially influence the performance of the qSOFA score. It is known, that altitude adaptation mechanisms can affect respiratory rate, oxygen saturation and blood pressure [41], and therefore alter the normal ranges for these parameters. Since large populations of around 389 million people live in altitudes above 1500 m [42], e. g. more than 8 million at >2300 m in Mexico City, further research on the influence of physiological adaptation mechanisms and reference values for these populations is warranted.

Compared to the national prevalence of people living with HIV/AIDS of 1.4%, the prevalence of HIV-infected patients of 11.8% within the study population was remarkably higher, as also previously reported from other sepsis cohorts from SSA [33, 43].

Almost one quarter of the study participants was considered underweight. Data from the general population for a comparison were not available, although clinical signs of malnutrition and low body mass index (<20 kg/m^2^) are common in the general population [44]. Malnourishment has not been evaluated as independent risk factor in other sepsis cohort investigations from SSA, but severe malnourishment seems to be independently associated with increased mortality [45]. In our study, we found no association between mortality rate and known HIV infection or nutritional status.

## 5. Conclusions

Sepsis is common and associated with high mortality in the study center, but remains underdiagnosed in clinical practice. The SOFA score was associated with mortality but is invasive and elaborate to assess and thus not suitable for routine use in LMIC. The easily applicable qSOFA score showed a poor performance for diagnosis of sepsis in comparison with other sepsis cohorts, having a higher sensitivity and markedly lower specificity in this cohort. The reasons for this finding warrant further investigation, including the role of adaptation mechanisms to high altitude.

Blood culture diagnostics proved cumbersome, requiring rigorous quality control mechanisms. Gram-negative bacteremia was significantly associated with a high mortality rate, with Gram-negative isolates showing a high rate of extensive AMR and an ineffectiveness of empirically administered antibiotic therapy.

Our results indicate the need for comprehensive evaluation of existing clinical tools for the diagnosis of sepsis in resource-limited settings and describe some of the options for optimization. Furthermore, it should be considered that diagnosing sepsis is only one of many aspects necessary for the adequate management of this complex clinical syndrome. Apart from the knowledge on the spectrum and resistance pattern of bacterial pathogens, improved treatment options for patients with severe sepsis and multi-organ failure are required. Finally, comprehensive capacity building and research activities are essential in order to raise awareness of sepsis, to understand the specific characteristics of sepsis and to identify and investigate potential intervention opportunities.

## Supporting information

S1 Data (SAV)

## References

[1] Cheng AC, West TE, Limmathurotsakul D, Peacock SJ. Strategies to reduce mortality from bacterial sepsis in adults in developing countries. PLoS Med. 2008;5: e175. 10.1371/journal.pmed.0050175 18752342 PMC2517616

[2] Cummings MJ, Goldberg E, Mwaka S, Kabajaasi O, Vittinghoff E, Cattamanchi A, et al. A complex intervention to improve implementation of World Health Organization guidelines for diagnosis of severe illness in low-income settings: a quasi-experimental study from Uganda. Implement Sci IS. 2017;12: 126. 10.1186/s13012-017-0654-0 29110667 PMC5674818

[3] Singer M, Deutschman CS, Seymour CW, Shankar-Hari M, Annane D, Bauer M, et al. The Third International Consensus Definitions for Sepsis and Septic Shock (Sepsis-3). JAMA. 2016;315: 801–810. 10.1001/jama.2016.0287 26903338 PMC4968574

[4] Rhodes A, Evans LE, Alhazzani W, Levy MM, Antonelli M, Ferrer R, et al. Surviving Sepsis Campaign: International Guidelines for Management of Sepsis and Septic Shock: 2016. Crit Care Med. 2017;45: 486–552. 10.1097/CCM.0000000000002255 28098591

[5] Vincent JL, de Mendonça A, Cantraine F, Moreno R, Takala J, Suter PM, et al. Use of the SOFA score to assess the incidence of organ dysfunction/failure in intensive care units: results of a multicenter, prospective study. Working group on “sepsis-related problems” of the European Society of Intensive Care Medicine. Crit Care Med. 1998;26: 1793–1800. 10.1097/00003246-199811000-00016 9824069

[6] McGloughlin S, Richards GA, Nor MBM, Prayag S, Baker T, Amin P. Sepsis in tropical regions: Report from the task force on tropical diseases by the World Federation of Societies of Intensive and Critical Care Medicine. J Crit Care. 2018;46: 115–118. 10.1016/j.jcrc.2017.12.018 29310974

[7] Schultz MJ, Dunser MW, Dondorp AM, Adhikari NKJ, Iyer S, Kwizera A, et al. Current challenges in the management of sepsis in ICUs in resource-poor settings and suggestions for the future. Intensive Care Med. 2017;43: 612–624. 10.1007/s00134-017-4750-z 28349179

[8] Seymour CW, Liu VX, Iwashyna TJ, Brunkhorst FM, Rea TD, Scherag A, et al. Assessment of Clinical Criteria for Sepsis: For the Third International Consensus Definitions for Sepsis and Septic Shock (Sepsis-3). JAMA. 2016;315: 762–774. 10.1001/jama.2016.0288 26903335 PMC5433435

[9] Lamontagne F, Harrison DA, Rowan KM. qSOFA for Identifying Sepsis Among Patients With Infection. JAMA. 2017;317: 267–268. 10.1001/jama.2016.19684 28114531

[10] Laxminarayan R, Matsoso P, Pant S, Brower C, Røttingen J-A, Klugman K, et al. Access to effective antimicrobials: a worldwide challenge. Lancet Lond Engl. 2016;387: 168–175. 10.1016/S0140-6736(15)00474-2 26603918

[11] Vernet G, Mary C, Altmann DM, Doumbo O, Morpeth S, Bhutta ZA, et al. Surveillance for antimicrobial drug resistance in under-resourced countries. Emerg Infect Dis. 2014;20: 434–441. 10.3201/EID2003.121157 24564906 PMC3944851

[12] Otu A, Elston J, Nsutebu E. Sepsis in Africa: practical steps to stem the tide. Pan Afr Med J. 2015;21: 323. 10.11604/pamj.2015.21.323.6462 26587170 PMC4633776

[13] Baelani I, Jochberger S, Laimer T, Otieno D, Kabutu J, Wilson I, et al. Availability of critical care resources to treat patients with severe sepsis or septic shock in Africa: a self-reported, continent-wide survey of anaesthesia providers. Crit Care Lond Engl. 2011;15: R10. 10.1186/cc9410 21219619 PMC3222039

[14] Stevenson EK, Rubenstein AR, Radin GT, Wiener RS, Walkey AJ. Two decades of mortality trends among patients with severe sepsis: a comparative meta-analysis*. Crit Care Med. 2014;42: 625–631. 10.1097/CCM.0000000000000026 24201173 PMC4313930

[15] Dellinger RP, Levy MM, Rhodes A, Annane D, Gerlach H, Opal SM, et al. Surviving sepsis campaign: international guidelines for management of severe sepsis and septic shock: 2012. Crit Care Med. 2013;41: 580–637. 10.1097/CCM.0b013e31827e83af 23353941

[16] Amir A, Saulters KJ, Olum S, Pitts K, Parsons A, Churchill C, et al. Outcomes of patients with severe sepsis after the first 6 hours of resuscitation at a regional referral hospital in Uganda. J Crit Care. 2016;33: 78–83. 10.1016/j.jcrc.2016.01.023 26994777

[17] Bebell LM, Ngonzi J, Bazira J, Fajardo Y, Boatin AA, Siedner MJ, et al. Antimicrobial-resistant infections among postpartum women at a Ugandan referral hospital. PloS One. 2017;12: e0175456. 10.1371/journal.pone.0175456 28406949 PMC5391058

[18] Thaver D, Ali SA, Zaidi AKM. Antimicrobial resistance among neonatal pathogens in developing countries. Pediatr Infect Dis J. 2009;28: S19–21. 10.1097/INF.0b013e3181958780 19106758

[19] Hamer DH, Darmstadt GL, Carlin JB, Zaidi AKM, Yeboah-Antwi K, Saha SK, et al. Etiology of bacteremia in young infants in six countries. Pediatr Infect Dis J. 2015;34: e1–8. 10.1097/INF.0000000000000549 25389919 PMC4272225

[20] Kabwe M, Tembo J, Chilukutu L, Chilufya M, Ngulube F, Lukwesa C, et al. Etiology, Antibiotic Resistance and Risk Factors for Neonatal Sepsis in a Large Referral Center in Zambia. Pediatr Infect Dis J. 2016;35: e191–198. 10.1097/INF.0000000000001154 27031259

[21] Majangara R, Gidiri MF, Chirenje ZM. Microbiology and clinical outcomes of puerperal sepsis: a prospective cohort study. J Obstet Gynaecol J Inst Obstet Gynaecol. 2018;38: 635–641. 10.1080/01443615.2017.1399112 29447024

[22] Dekker D, Krumkamp R, Eibach D, Sarpong N, Boahen KG, Frimpong M, et al. Characterization of Salmonella enterica from invasive bloodstream infections and water sources in rural Ghana. BMC Infect Dis. 2018;18: 47. 10.1186/s12879-018-2957-4 29351771 PMC5775569

[23] Kajumbula H, Fujita AW, Mbabazi O, Najjuka C, Izale C, Akampurira A, et al. Antimicrobial Drug Resistance in Blood Culture Isolates at a Tertiary Hospital, Uganda. Emerg Infect Dis. 2018;24: 174–175. 10.3201/eid2401.171112 29260682 PMC5749445

[24] Musicha P, Cornick JE, Bar-Zeev N, French N, Masesa C, Denis B, et al. Trends in antimicrobial resistance in bloodstream infection isolates at a large urban hospital in Malawi (1998–2016): a surveillance study. Lancet Infect Dis. 2017;17: 1042–1052. 10.1016/S1473-3099(17)30394-8 28818544 PMC5610140

[25] Bone RC, Balk RA, Cerra FB, Dellinger RP, Fein AM, Knaus WA, et al. Definitions for sepsis and organ failure and guidelines for the use of innovative therapies in sepsis. The ACCP/SCCM Consensus Conference Committee. American College of Chest Physicians/Society of Critical Care Medicine. Chest. 1992;101: 1644–1655. 10.1378/chest.101.6.1644 1303622

[26] Rhodes A, Evans LE, Alhazzani W, Levy MM, Antonelli M, Ferrer R, et al. Surviving Sepsis Campaign: International Guidelines for Management of Sepsis and Septic Shock: 2016. Crit Care Med. 2017;45: 486–552. 10.1097/CCM.0000000000002255 28098591

[27] Strauß LM, Dahms C, Becker K, Kramer A, Kaase M, Mellmann A. Development and evaluation of a novel universal β-lactamase gene subtyping assay for blaSHV, blaTEM and blaCTX-M using clinical and livestock-associated Escherichia coli. J Antimicrob Chemother. 2015;70: 710–715. 10.1093/jac/dku450 25414200

[28] Wendel AF, Brodner AHB, Wydra S, Ressina S, Henrich B, Pfeffer K, et al. Genetic Characterization and Emergence of the Metallo-β-Lactamase GIM-1 in Pseudomonas spp. and Enterobacteriaceae during a Long-Term Outbreak. Antimicrob Agents Chemother. 2013;57: 5162–5165. 10.1128/AAC.00118-13 23877696 PMC3811479

[29] Park HK, Kim WY, Kim MC, Jung W, Ko BS. Quick sequential organ failure assessment compared to systemic inflammatory response syndrome for predicting sepsis in emergency department. J Crit Care. 2017;42: 12–17. 10.1016/j.jcrc.2017.06.020 28647650

[30] Finkelsztein EJ, Jones DS, Ma KC, Pabón MA, Delgado T, Nakahira K, et al. Comparison of qSOFA and SIRS for predicting adverse outcomes of patients with suspicion of sepsis outside the intensive care unit. Crit Care Lond Engl. 2017;21: 73. 10.1186/s13054-017-1658-5 28342442 PMC5366240

[31] Kaukonen K-M, Bailey M, Suzuki S, Pilcher D, Bellomo R. Mortality related to severe sepsis and septic shock among critically ill patients in Australia and New Zealand, 2000–2012. JAMA. 2014;311: 1308–1316. 10.1001/jama.2014.2637 24638143

[32] Stoller J, Halpin L, Weis M, Aplin B, Qu W, Georgescu C, et al. Epidemiology of severe sepsis: 2008–2012. J Crit Care. 2016;31: 58–62. 10.1016/j.jcrc.2015.09.034 26601855

[33] Waitt PI, Mukaka M, Goodson P, SimuKonda FD, Waitt CJ, Feasey N, et al. Sepsis carries a high mortality among hospitalised adults in Malawi in the era of antiretroviral therapy scale-up: A longitudinal cohort study. J Infect. 2015;70: 11–19. 10.1016/j.jinf.2014.07.004 25043393 PMC4291151

[34] Jacob ST, Moore CC, Banura P, Pinkerton R, Meya D, Opendi P, et al. Severe Sepsis in Two Ugandan Hospitals: a Prospective Observational Study of Management and Outcomes in a Predominantly HIV-1 Infected Population. PLoS ONE. 2009;4. 10.1371/journal.pone.0007782 19907656 PMC2771355

[35] Cheng MP, Stenstrom R, Paquette K, Stabler SN, Akhter M, Davidson AC, et al. Blood Culture Results Before and After Antimicrobial Administration in Patients With Severe Manifestations of Sepsis: A Diagnostic Study. Ann Intern Med. 2019. 10.7326/M19-1696 31525774

[36] Zeynudin A, Pritsch M, Schubert S, Messerer M, Liegl G, Hoelscher M, et al. Prevalence and antibiotic susceptibility pattern of CTX-M type extended-spectrum β-lactamases among clinical isolates of gram-negative bacilli in Jimma, Ethiopia. BMC Infect Dis. 2018;18: 524. 10.1186/s12879-018-3436-7 30342476 PMC6196031

[37] Pritsch M, Zeynudin A, Messerer M, Baumer S, Liegl G, Schubert S, et al. First report on bla NDM-1-producing Acinetobacter baumannii in three clinical isolates from Ethiopia. BMC Infect Dis. 2017;17: 180. 10.1186/s12879-017-2289-9 28249575 PMC5333390

[38] Dorsett M, Kroll M, Smith CS, Asaro P, Liang SY, Moy HP. qSOFA Has Poor Sensitivity for Prehospital Identification of Severe Sepsis and Septic Shock. Prehospital Emerg Care Off J Natl Assoc EMS Physicians Natl Assoc State EMS Dir. 2017;21: 489–497. 10.1080/10903127.2016.1274348 28121217

[39] Askim Å, Moser F, Gustad LT, Stene H, Gundersen M, Åsvold BO, et al. Poor performance of quick-SOFA (qSOFA) score in predicting severe sepsis and mortality—a prospective study of patients admitted with infection to the emergency department. Scand J Trauma Resusc Emerg Med. 2017;25: 56. 10.1186/s13049-017-0399-4 28599661 PMC5466747

[40] Williams JM, Greenslade JH, McKenzie JV, Chu K, Brown AFT, Lipman J. Systemic Inflammatory Response Syndrome, Quick Sequential Organ Function Assessment, and Organ Dysfunction: Insights From a Prospective Database of ED Patients With Infection. Chest. 2017;151: 586–596. 10.1016/j.chest.2016.10.057 27876592

[41] Beall CM. Two routes to functional adaptation: Tibetan and Andean high-altitude natives. Proc Natl Acad Sci U S A. 2007;104 Suppl 1: 8655–8660. 10.1073/pnas.0701985104 17494744 PMC1876443

[42] Cohen JE, Small C. Hypsographic demography: the distribution of human population by altitude. Proc Natl Acad Sci U S A. 1998;95: 14009–14014. 10.1073/pnas.95.24.14009 9826643 PMC24316

[43] Rudd KE, Tutaryebwa LK, West TE. Presentation, management, and outcomes of sepsis in adults and children admitted to a rural Ugandan hospital: A prospective observational cohort study. PloS One. 2017;12: e0171422. 10.1371/journal.pone.0171422 28199348 PMC5310912

[44] Alemu T, Lindtjørn B. Physical activity, illness and nutritional status among adults in a rural Ethiopian community. Int J Epidemiol. 1995;24: 977–983. 10.1093/ije/24.5.977 8557456

[45] Irving SY, Daly B, Verger J, Typpo KV, Brown A-M, Hanlon A, et al. The association of nutrition status expressed as body mass index z-score with outcomes in children with severe sepsis: a secondary analysis from the Sepsis Prevalence, Outcomes and Therapies (SPROUT) study. Crit Care Med. 2018;46: e1029–e1039. 10.1097/CCM.0000000000003351 30095495 PMC6185775

